# Study protocol: safety and efficacy of propranolol in newborns with Retinopathy of Prematurity (PROP-ROP): ISRCTN18523491

**DOI:** 10.1186/1471-2431-10-83

**Published:** 2010-11-18

**Authors:** Luca Filippi, Giacomo Cavallaro, Patrizio Fiorini, Marta Daniotti, Valentina Benedetti, Gloria Cristofori, Gabriella Araimo, Luca Ramenghi, Agostino La Torre, Pina Fortunato, Liliana Pollazzi, Giancarlo la Marca, Sabrina Malvagia, Paola Bagnoli, Chiara Ristori, Massimo Dal Monte, Anna Rita Bilia, Benedetta Isacchi, Sandra Furlanetto, Francesca Tinelli, Giovanni Cioni, Gianpaolo Donzelli, Silvia Osnaghi, Fabio Mosca

**Affiliations:** 1 Neonatal Intensive Care Unit, Department of Perinatal Medicine, "A. Meyer" University Children's Hospital, Florence, Italy; 2 Institute of Pediatrics and Neonatology, Department of Maternal and Pediatric Sciences, Fondazione IRCCS Cà Granda Ospedale Maggiore Policlinico, University of Milan, Milan, Italy; 3 Department of Specialised Surgical Sciences, University of Florence, Italy; 4 Pediatric Ophthalmology Unit, "A. Meyer" University Children's Hospital, Florence, Italy; 5 Neurometabolic Unit, Department of Pediatric Neurosciences, "A. Meyer" University Children's Hospital, Florence, Italy; 6 Department of Biology, Unit of General Physiology, University of Pisa, Pisa, Italy; 7 Department of Pharmaceutical Sciences, University of Florence, Italy; 8 Department of Developmental Neuroscience, IRCCS Stella Maris, Calambrone, Pisa, Italy; 9 Division of Child Neurology and Psychiatry, University of Pisa, Pisa, Italy; 10 Department of Perinatal Medicine, University of Florence, Italy; 11 Ophthalmology Unit, Department of Neuroscience, Fondazione IRCCS Cà Granda Ospedale Maggiore Policlinico, University of Milan, Milan, Italy

## Abstract

**Background:**

Despite new therapeutic approaches have improved the prognosis of newborns with retinopathy of prematurity (ROP), an unfavourable structural and functional outcome still remains high. There is high pressure to develop new drugs to prevent and treat ROP. There is increasing enthusiasm for anti-VEGF drugs, but angiogenic inhibitors selective for abnormal blood vessels would be considered as an optimal treatment.

In an animal experimental model of proliferative retinopathy, we have recently demonstrated that the pharmacological blockade of beta-adrenoreceptors improves retinal neovascularization and blood retinal barrier breakdown consequent to hypoxia. The purpose of this study is to evaluate the propranolol administration in preterm newborns suffering from a precocious phase of ROP in terms of safety and efficacy in counteracting the progression of retinopathy.

**Methods/Design:**

Preterm newborns (gestational age at birth lower than 32 weeks) with stage 2 ROP (zone II-III without plus) will be randomized, according to their gestational age, to receive propranolol added to standard treatment (treatment adopted by the ETROP Cooperative Group) or standard treatment alone. Propranolol will be administered until retinal vascularization will be completely developed, but not more than 90 days. Forty-four participants will be recruited into the study. To evaluate the safety of propranolol administration, cardiac and respiratory parameters will be continuously monitored. Blood samplings will be performed to check renal, liver and metabolic balance. To evaluate the efficacy of propranolol, the progression of the disease, the number of laser treatments or vitrectomies, the incidence of retinal detachment or blindness, will be evaluated by serial ophthalmologic examinations. Visual function will be evaluated by means of behavioural standardized tests.

**Discussion:**

This pilot study is the first research that explores the possible therapeutic role of beta blockers in ROP. The objective of this research is highly ambitious: to find a treatment simple, inexpensive, well tolerated and with few adverse effects, able to counteract one of the major complications of the prematurity. Any favourable results of this research could open new perspectives and original scenarios about the treatment or the prevention of this and other proliferative retinopathies.

**Trial Registration:**

Current Controlled Trials ISRCTN18523491; ClinicalTrials.gov Identifier NCT01079715; EudraCT Number 2010-018737-21

## Background

### The retinopathy of prematurity

#### A. Disease incidence

Retinopathy of prematurity (ROP) is a major cause of blindness and visual impairment in children in both developing and developed countries around the world, despite of progressive improvements in neonatal care [1]. The overall incidence of any ROP in the USA varies from 65 [2] to 68% [3] among infants with a birth weight less than 1,250 g. However, the overall incidence of more-severe ROP (prethreshold), a condition that can lead to retinal detachment and blindness, is progressively increased to around thirty-seven percent among infants with ROP in the ETROP Study [3]. The incidence of this disease is closely related to the birth weight and the gestational age at birth: the lower the birth weight and earlier postconceptional age at birth, the higher the likelihood of developing a more severe disease. However, preterm infants developing severe ROP in middle and low income countries have a wider range of birth weights and gestational ages than what is usually observed in industrialized countries [1].

#### B. Disease pathogenesis

ROP is a multifactorial neovascularizing disease that affects premature infants, characterized by perturbation of the normal vascular development of the retina. In the human fetus, retinal blood vessel development begins during the fourth month of gestation, and this process usually occurs in the hypoxic uterine environment. Therefore, in very premature infants, the retina is nearly avascular at birth, and premature birth usually stops the process of retinal vascular development that normally occurs in the hypoxic uterine environment [4].

The pathogenesis of ROP is hypothesized to consist of two distinct phases [5]. The exposure to extra-uterine relative hyperoxia amplified by supplemental oxygen delivery retards or blocks the normal retinal vascular growth (first phase of ROP), decreasing the Vascular Endothelial Growth Factor (VEGF) expression and endothelial cell proliferation [6]. The loss of placenta contributes to reduce the vascularization of retina due to the reduction of the Insulin-like Growth Factor-1 (IGF-1) levels (largely produced by the placenta) [7]. Therefore, this first phase of ROP is characterized by cessation of vessel growth and loss of vessels.

The second phase of ROP begins at 32-34 weeks of postmenstrual age, and is characterized by a hypoxia-induced retinal neovascularization similar to that observed in other proliferative retinopathies such as diabetic retinopathy or age-related macular degeneration [4].

The shift to this proliferative phase of ROP is usually explained by the imbalance between the poorly developed blood vessels and the increasing metabolic demands of developing neural retina. This imbalance produces retinal hypoxia, that increases the stability of inducible α subunit of the transcription factor hypoxia-inducible factor (HIF)-1. HIF-1α accumulation leads to the subsequent transactivation of HIF which, in turn, upregulates the expression of a variety of genes including those encoding for angiogenic growth factors [8]. Among them, VEGF, IGF-1, and their receptors induce a pathological blood vessel formation at the junction between the vascularized retina and the avascular zone of the retina, also into the vitreous. Progressively, this pathological neovascularization produces a fibrous scar extending from the retina to the vitreous gel and lens, the retraction of which can separate the retina from the retinal pigment epithelium, resulting in a retinal detachment and likely blindness [4].

However this explanation of the shift from the first to the second phase of ROP is rather indefinite. All contributions capable to better explain this transition could have important implications for the understanding and treatment of ROP.

#### C. VEGF in retinopathy of prematurity

Oxygen tension plays a key role in VEGF expression. Hyperoxia decreases VEGF levels, and this phenomenon has been hypothesized to play a key role in the first stage of ROP. Precocious exposure to extra-uterine relative hyperoxia, as observed in premature newborns, results in suppression of VEGF expression, increased apoptosis and vasoattenuation [9,10].

Instead, hypoxia is the driving force for blood vessel growth, through the increase in VEGF gene expression [11-13], as observed during the second phase of ROP, which is a proliferative phase.

VEGF is now considered essential in driving the development and growth of blood vessels [14]. Increased VEGF expression is seen in Müller cells and astrocytes of the inner retina during the development of neovascularization [15], and an increase in VEGF receptors is observed in the vicinity of target endothelial cells [16,17]. VEGF is overexpressed in response to hypoxia and ischemia. As hypoxia is relieved by oxygen release from the newly formed vessels, then VEGF overexpression is drastically reduced. VEGF stimulates its receptor VEGFR-2 and induces endothelial cell cytoprotection through the activation of the protein kinase B (PKB)/Akt pathway [18], whereas the stimulation of mitogen-activated protein kinase (MAPK) cascade by VEGF/VEGFR-2 promotes the proliferation of endothelial cells [19].

#### D. Role of IGF-1

In mice, IGF-1 is critical for physiological development of retinal vessels: the lack of IGF-1 in IGF-1 knockout mice, in fact, depresses blood vessel development, despite the presence of VEGF [20].

Low levels of IGF-1 prevent VEGF-induced activation of PKB/Akt, a critical kinase for endothelial cell survival [20]. In premature newborns, levels of IGF-1 have been reported to be low, as a consequence of loss of the placenta, and ROP risk has been associated with low circulating levels of IGF-1 [21]. In fact serial measurements of serum IGF-1 levels in preterm newborns have demonstrated that IGF-1 levels are inversely correlated with the severity of clinical ROP [7,21,22]. Low levels of IGF-1 compromised both endothelial cell survival and proliferation pathways. These data suggest that low IGF-1 levels could contribute to the vessels degeneration typical of the first phase of ROP [21].

However, IGF-1 also plays an important role also in the proliferative phase of ROP. With maturation, IGF-1 increases slowly, reaching a threshold level at around 34 weeks of gestational age. Then, IGF-1 promotes VEGF-induced neovascularization, most likely through the stimulation of the MAPK pathway [23].

Similarly to what observed for VEGF, the mechanism by which IGF-1 progressively increases is, to date, still unknown.

#### E. Role of oxygen

Studies in preterm infants have demonstrated a relation between exposure to high levels of oxygen and the development of ROP [24-27]. The high extrauterine concentration of oxygen suppresses VEGF expression, and this phenomenon explains the vaso-obliteration induced by the apoptosis of vascular endothelial cells in the first phase of ROP.

Actually, the idea that relative hypoxia of avascular retina is responsible for the development of the second phase of ROP could be explained by the hypoxia-driven production of VEGF, although this mechanism does not explain the increase of IGF-1, that is usually considered a non-oxygen-regulated factor [7].

This theory raised the possibility that supplemental oxygen might be used to improve retinal oxygenation and to down-regulate retinal neovascularization. The STOP-ROP Study has been planned to test the hypothesis that supplemental oxygen administered to preterm infants with pre-threshold ROP would improve retinal oxygenation, down-regulate retinal neovascularization, and finally reduce the progression of ROP. However, newborns randomized to receive oxygen to keep their oxygen saturations either 88-94% or 96-99%, did not show any differences in the progression of ROP [28].

#### F. Conclusion

Until now, a clear explanation of the progression from the first to the second phase of ROP has not been given. Probably a better understanding of this passage may provide new therapeutic possibilities.

#### G. Standard treatment of retinopathy of prematurity

The best currently available management of ROP consists of the early identification of high-risk vascular pattern of the developing retina (i.e., dilated venules and tortuous arterioles at the posterior pole of the eye) at first, and then in the laser ablation of the avascular peripheral retina. Multicenter trials have indicated the manner in which ROP should be monitored and treated. The CRYO-ROP studies showed that treating threshold diseases (stage 3 ROP in at least five contiguous or eight non-contiguous clock hours) improved visual and structural outcomes in premature infants [29,30]. Benefits of earlier treatment were investigated in the ETROP study [31]. This study demonstrated that ablative therapy was beneficial for any eye with any stage of ROP in zone I with plus disease, stage 3 ROP in zone I with or without plus disease, and stage 2 or 3 ROP in zone II with plus disease [32]. The 2-year data collected in the ETROP study showed that unfavourable structural outcomes (defined as retinal folds or detachment) decreased in eyes that received early ablative therapy [33]. Visual acuity improved at 6 years of age only in newborns with Type 1 high-risk prethreshold eyes [34] (those with plus disease in either Zone I or Zone II, or Zone I stage 3 disease) [32]. Long-term structural and functional outcomes suggest that laser photocoagulation is superior to cryotherapy [35,36].

Despite these advances, the unfavourable structural and functional outcomes still remain high, and treatment with laser photocoagulation carries itself a certainty of lasting peripheral visual dysfunction.

#### H. Novel treatments of retinopathy of prematurity

Since ROP is a VEGF-dependent vasoproliferative disease, it is not surprising that there is increasing enthusiasm for anti-VEGF drugs (bevacizumab, ranibizumab) [37-39]. Anti-VEGF drugs do not ablate retinal tissue, and seem to be effective to counteract the abnormal neovascularization of the ridge (the demarcation line between vascularized and avascular retina characteristic of stage 2 ROP). However, undesirable consequences could be met because VEGF and its receptors play an important role in the development of the neural retina, and potential adverse effects of anti-VEGF drugs can not be excluded on developing neurons in the immature retina [4]. For instance, mice treated with specific VEGF receptor antagonists demonstrate a cell loss in the inner nuclear layer, containing Muller cell nuclei and in the ganglion cell layer, containing astrocytes [40].

In addition, the dosage and the timing of injections are still to be defined. Moreover, the incidence of complications related to the need of multiple injections including ocular trauma or endophthalmitis, together with the systemic effects of such antagonists, are still to be evaluated.

Consequently, new pharmacological approaches for the prevention and treatment of ROP are urgently needed. The ideal treatment would be to find an angiogenic inhibitor selective for abnormal blood vessels, but this drug is yet to be developed.

#### I. Beta-adrenoreceptor function and vascularization

Recently, propranolol, a well-tolerated, non-selective, beta-adrenoreceptor (beta-AR) blocker has been reported as an effective drug in reducing the growth of infantile hemangiomas, the most common tumour of infancy [41]. It has been hypothesized that propranolol may act through a reduction of VEGF levels [42].

There is some evidence that the adrenergic system is involved in the regulation of hypoxia-induced neovascularization and VEGF production. For instance, in embryonic heart, hypoxia has been shown to cause catecholaminergic overstimulation that, in turn, alters signalling pathways associated with beta-adrenoreceptors (ARs) [43]. In addition, norepinephrine stimulates the production of VEGF in endothelial cells of the human umbilical vein, [44], and induces vascular VEGF gene expression in brown adipocytes [45]. The role of the adrenergic system in the regulation of proangiogenic factors has also been demonstrated in solid tumours and tumour cell lines: norepinephrine, in fact, affects tumour progression by upregulating VEGF through the beta_2_-AR stimulation. This proangiogenic effect is antagonized by propranolol [46-54].

Beta_2_-ARs are widely expressed on vascular endothelial cells [55], and an interesting study provided evidence that beta_2_-ARs can regulate neoangiogenesis in response to chronic ischemia. In fact, in the endothelium of the rat femoral artery, ischemia produces a beta_2_-ARs overexpression on endothelial cells promoting VEGF production, cell proliferation, and function including revascularization. This observation suggests a novel and physiologically relevant role of beta_2_-ARs in neoangiogenesis in response to ischemia [56].

**Figure 1:**
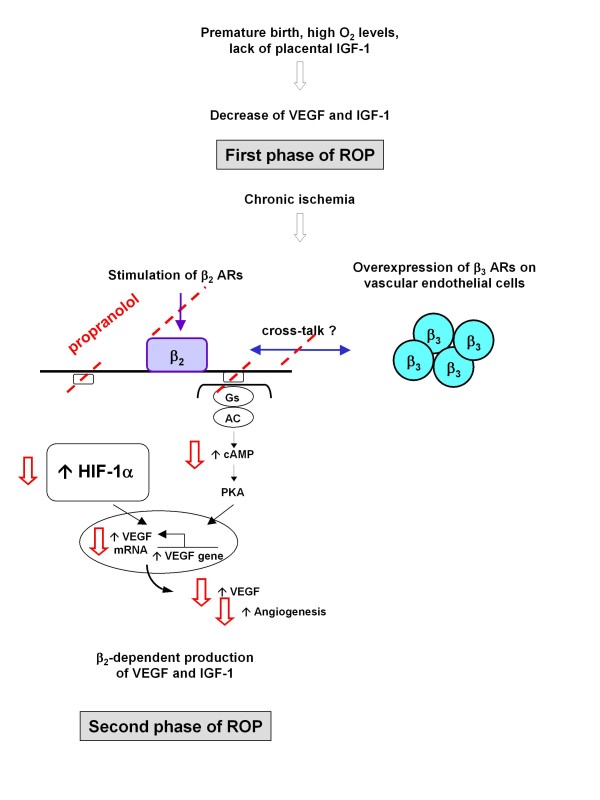
**Beta_2_-adrenoreceptors stimulation and ROP: role of propranolol in pharmacological blockade of abnormal neovascularization**. Red arrows show the likely effects of propranolol.

Considering that beta_2_-AR stimulation upregulates VEGF, and that the second phase of ROP is supported by an increased VEGF production, we hypothesized that VEGF overexpression in ROP could be induced by beta_2_-ARs stimulation, and that beta-blockers could be useful in the treatment of ROP (Figure 1). This hypothesis was supported by the observation that infantile hemangiomas are associated with the development of ROP in infants, suggesting a possible pathogenic relationship between the two diseases [57].

Beta-AR expression in the retina has been established at both the messenger and the protein level [58]. In addition, distinct beta-ARs have been localized in cultured retinal endothelial cells, Muller cells and retinal pigment epithelium [59-62]. Little is known about beta-AR localization and function in the retina [61,63]. Beta_3_-AR activation has been reported to cause proliferation and migration of retinal endothelial cells [59]. In addition, in human choroidal endothelial cells, the beta-AR agonist isoproterenol leads to increased level of growth factors implicated in ocular diseases [60].

#### J. New research perspectives derived from recent studies on oxygen-induced retinopathy model

Recently we tested the role of the adrenergic system in a mouse model of oxygen-induced retinopathy (OIR) [64], considering that this animal model is characterized by the abnormal formation of new blood vessels which can be assimilated to ROP [65]. We investigated the role of propranolol in regulation of retinal angiogenesis and vascular permeability. More specifically, we determined whether propranolol affects retinal levels of proangiogenic factors, vascular leakage and retinal neovascularization. The mechanisms of action of propranolol on angiogenesis were also investigated.

We demonstrated that, in OIR mice, the retinal expression of HIF-1α significantly increased (about 7.5 fold), as compared to control littermates. Hypoxia upregulated VEGF, VEGF receptor 1 (VEGFR-1), VEGF receptor 2 (VEGFR-2), IGF-1 and IGF-1 receptor (IGF-1R) messengers as well as VEGF protein. Moreover, hypoxia increased beta_3_-AR protein with dense beta_3_-AR-immunoreactivity localized to engorged retinal tufts.

Treatment with propranolol partially restored the hypoxia-induced increase in IGF-1 mRNA and VEGF mRNA. Hypoxic levels of VEGF protein were dose-dependently reduced by propranolol, without affecting those of VEGFR-1, VEGFR-2 and IGF1-R mRNAs.

Mechanisms coupling beta-AR blockade with VEGF inhibition included an important role of the transcription factor HIF-1α, which was consistently reduced by propranolol, indicating that HIF-1α is likely to participate in the mechanisms coupling beta-AR blockade with VEGF inhibition. The additional finding that the IGF1-R antagonist picropodophyllin to propranolol-treated mice did not affect the propranolol-induced inhibition of retinal VEGF suggests that propranolol effects on VEGF did not involve IGF-1 signalling, although IGF-1 is a potent inducer of VEGF.

The pharmacological blockade of beta-ARs interferes with retinal neovascularization through propranolol-induced downregulation of proangiogenic factors. In fact, in our study, propranolol drastically reduced retinal haemorrhages and tufts, improved the retinopathy score, partially restored the levels of the tight junction protein occludin and decreased extravascular leakage of albumin. The efficacy of propranolol in reducing the hypoxia-induced blood retinal barrier breakdown was finally confirmed with the Evan's blue method [66].

Propranolol reduced VEGF overproduction in the hypoxic retina, but did not affect the VEGF levels in the normoxic retina, suggesting different pattern of regulation of VEGF transcriptional pathways in normoxic and hypoxic conditions. This possibility is supported by the additional finding that beta-AR blockade does not influence VEGF levels in the brain, lungs or heart in which VEGF expression is not regulated by hypoxia, indicating that these organs probably do not experience hypoxia in the OIR model. These data also suggest that only the VEGF produced under hypoxia-ischemia (most likely induced through the HIF-1α stimulation) can be affected by the inhibition of beta-ARs.

Moreover, we demonstrated that propranolol did not reduce vascular hyperpermeability induced by intravitreal injection of exogenous VEGF. This finding suggests that propranolol does not influence the VEGF-induced downstream signalling pathways, consistent with the result that the expression of VEGF receptors is not changed after propranolol treatment [66].

These results provide the first demonstration that beta-ARs are coupled with a modulation of VEGF and IGF-1 in the OIR model and suggest a role of catecholamines in the shift from the first to the second phase of ROP. The hypothesis on which we are currently working on, is that in this animal model the chronic ischemia induces an overexpression of beta_3_-ARs on vascular endothelial cells, which in turn could stimulate the activation (cross-talk) of beta_2_-ARs (data still unpublished).

### Hypothesis

Our finding that in mice hypoxic retinas propranolol downregulates proangiogenic factors, improves the proangiogenic effect of hypoxia, and repairs at least in part the hypoxia-induced blood retinal barrier breakdown is particularly intriguing in light of a possible therapeutic use of beta-AR blockers to counteract retinal neovascularization in ROP.

Our hypothesis is that also in human preterm newborns with ROP, VEGF overexpression could be induced by beta_2_-AR stimulation, and that propranolol, a well-tolerated, non-selective, beta-AR blocker, administered in preterm newborns when a precocious phase of ROP is detected, could reduce the progression of the disease.

### Objectives

#### Major objectives: safety and efficacy of propranolol

Propranolol in children is considered safe and generally well tolerated. Nevertheless, the possibility of some side effects has to be considered. Reported side effects are usually mild and transient, and include bronchospasm, heart failure, prolonged hypoglycaemia, bradycardia, heart block [67]. Such problems have also been observed also in newborns [68,69], but they may be clinically relevant in unstable preterm newborns.

The main purpose of this study is to evaluate the safety of propranolol administration in such premature newborns. To evaluate its safety, cardiac and respiratory parameters (heart frequency, blood pressure, oxygen saturation, respiratory support), will be continuously monitored. Blood samplings will be performed to check renal, liver and metabolic balance.

Further objective of this study is to evaluate the efficacy of propranolol to reduce the progression of ROP, the incidence of either retinal detachment or blindness, through serial ophthalmologic examinations, planned at different intervals according to the severity of ROP, in comparison with what observed in a control group receiving conventional treatment (treatment adopted by the ETROP Cooperative Group).

All newborns will be evaluated at 40 weeks of gestational age by using a recently published battery of behavioural tests designed to assess various aspects of visual function [70], which includes items that assess ocular movements (spontaneous behaviour and in response to a target), the ability to fix and follow a black/white target (horizontally, vertically, and in an arc), the reaction to a coloured target, the ability to discriminate between black and white stripes of increasing spatial frequency, and the ability to keep attention on a target that is moved slowly away from the infant. Visual function will be evaluated again at 1, 4 1/2, 12, 18 and 24 months corrected age [71] with particular regards to visual acuity (binocular and monocular), measured by means of well known instruments based on preferential force choice (Teller acuity cards), stereopsis and ocular motricity.

### Secondary objectives

1) plasma propranolol concentration will be determined through serial measurements on dried blood spots, to characterize its pharmacokinetic profile in relation to the clinical effects of the therapy. In the recent years the use of dried blood spot (DBS) technology to obtain pharmacokinetic data has increased [72,73]. DBS sampling has many advantages, which include reducing the volume of blood required from patients such as newborns with limited available quantity of blood. Other advantages include a less invasive sampling method, and easy, and cheap sample collection, transportation, and storage.

2) plasma concentrations of proangiogenic markers will be determined in premature newborns treated with propranolol and compared with those measured in premature infants with standard treatment, to investigate whether propranolol decreases their plasma levels. The aim is to evaluate the efficacy of propranolol in regulating the plasma levels of VEGF, the soluble forms of the tyrosine kinase receptors, sVEGFR-2 and sTie-2 and sE-selectin, an inducible endothelial leukocyte adhesion molecule expressed on the surface of endothelial cells [74,75].

## Methods/Design

### a. Study design

We planned an interventional pilot randomized controlled trial in two-centre to compare the safety and efficacy of propranolol associated to a conventional approach (treatment adopted by the ETROP Cooperative Group) versus conventional approach alone to treat preterm newborns (gestational age less than 32 weeks) with a stage 2 ROP (zone II-III without plus). Propranolol will be administered per os at the dose of 0.5 mg/kg/every 6 hours. The protocol provides that ophthalmologists will be blindfolded about which newborns will be treated with propranolol in addition to conventional approach.

### b. Experimental plan and data analysis

Based on the medical literature, the percentage of stage 2 ROP that progresses to more-severe ROP is around 36% [3]. We hypothesized that treatment with propranolol may be able to block the progression of this disease and therefore that the percentage of the progression to more severe ROP may be zero. In order to compare the proportions of newborns in propranolol group (treated) and control group (standard treatment), that progresses to more-severe ROP, the estimated sample size was calculated, considering normal distribution, an alpha error of 0.05 and a power of 80 percent. The sample size for each group is 22 participants.

The incidence of progression from stage 2 ROP to higher stages increases with the decreasing of the gestational age. To ensure a homogeneous distribution of the gestational age in both groups (treated and controls), the recruited newborns will be randomized and stratified according to their gestational age in three different groups: group 1 (23-25 weeks), group 2 (26-28 weeks), and group 3 (29-32 weeks)

The two units have an overall admission rate of approximately 300 very low birth weight babies per year. Due to the high hypothetical advantages of this treatment, we estimate 90-100% rate of consent and we predict we would recruit the 44 newborns over around 18 months period with a realistic safety margin.

To evaluate propranolol safety, cardiac and respiratory parameters (heart frequency, blood pressure, oxygen saturation, respiratory support), will be continuously monitored. Blood samplings will be performed as soon as the stage 2 ROP will be diagnosed, to check renal, liver and metabolic balance. Kruskal-Wallis test will be used to assess possible differences between newborns treated or not with propranolol. The safety will be also evaluated by means of relative risk (RR) [76]. RR will be calculated as the ratio between the probability of side effects in the propranolol group with respect to the control group.

RR will be also calculated as the ratio between the probability that ROP progresses to more-severe ROP in propranolol group with respect to the control group. In this case, values of RR lower than 1, will be associated to the efficacy of the treatment. If necessary, RR for each gestational age group, will be obtained.

### c. Allocation of participants to the trial groups (Randomization)

Randomization will be done stratifying eligible newborns for the gestational age (23-25, 26-28 and 29-32 weeks) to ensure identical incidence of risk. In both recruiting hospitals, newborns will be randomized in blocks of eight for each gestational age group, alternating between propranolol added to standard treatment and standard treatment alone, where standard treatment is the treatment adopted by the ETROP Cooperative Group [32].

### d. Study population-setting

Preterm newborns delivered at gestational age lower than 32 weeks admitted to the Neonatal Intensive Care Unit at the A. Meyer University Childrens' Hospital, Florence and at the Institute of Pediatrics and Neonatology, Fondazione IRCCS Ospedale Maggiore Policlinico, Mangiagalli e Regina Elena, University of Milan.

### e. Inclusion criteria

1. Preterm newborns (gestational age lower than 32 weeks) who developed stage 2 ROP (zone II-III without plus).

2. Informed Consent from a parent.

### f. Selection criteria for study subjects

At least one of the parents of newborns who meet the inclusion criteria will be approached by the study investigator/nurse and informed of the study. A signed parental informed consent will be obtained.

### g. Exclusion criteria

1. Newborns with congenital cardiovascular anomalies, renal failure, failure to thrive, cerebral haemorrhage, which contraindicate the use of beta-blockers.

2. Newborns with ROP at a more advanced stage than stage 2 (zone II-III without plus).

3. Newborns in whom propranolol administration will be stopped for more than two doses, with the exception of a temporary suspension before surgery.

4. Informed Consent from a parent refused.

### h. Stop criteria

The study could be stopped if severe hypotension, bradycardia or bronchospasm develops; in this case, the opportunity to reduce the dose of propranolol may be considered.

### i. Special condition

Taking into account that newborns with posthemorrhagic hydrocephalus usually have high levels of VEGF in the cerebrospinal fluid [77,78], such neonates will be enrolled, but analyzed separately.

### j. Ethical approval

A two-centre phase II pilot study entitled "Safety and efficacy of propranolol in newborns with Retinopathy of Prematurity (PROP-ROP): a pilot study" has been approved by the Ethics Committees of both A. Meyer University Childrens' Hospital, Florence and Fondazione IRCCS Cà Granda Ospedale Maggiore Policlinico, Milan.

Informed consent will be obtained from at least one of the parents prior to study entry. Parents will be given full verbal and written information regarding the objective and procedures of the study and the possible risks involved.

Propranolol is considered safe for use in children. Nevertheless, a careful watch will be kept on all study participants with regard to side effects of drug administration. Parents of participants will be made aware of possible side effects. Infants will be monitored in the Neonatal Intensive Care Unit throughout the study period and their clinical condition will be evaluated daily as part of medical rounds. A letter informing the participant and the family doctor as to which study arm the participant had been randomized to will be sent following completion of the study.

In the presence of adverse events, a reduction of dosage will be taken into account.

For ethical and scientific reasons, an interim analysis after the enrolment of half of the newborns, is planned.

### k. Duration of the treatment

The treatment period will begin following randomization. On day 0 baseline measurements will be taken and recorded, and propranolol administration will be begun. This treatment will continue until the complete development of retinal vascularization, although this administration can not last more than 90 days.

### l. Follow-up

Ophthalmologic evaluations are planned usually every 3-4 days, or more frequently according to clinical evolution and severity of ROP, until the end of the vascularization process is achieved. Ophthalmologists will be blindfolded about which newborns have been treated with propranolol in addition to conventional approach.

The efficacy will be evaluated comparing the different incidences of the progression of ROP to stages 3 or to retinal detachment, the different incidence of laser treatment, the different incidence of vitrectomy, between the two groups. To evaluate structural and functional outcome, the follow-up is planned at 40 weeks gestational age, 4 1/2, 12, 18 and 24 months corrected age.

### m. Measurement of outcomes

#### 1. Primary endpoint

To evaluate if the propranolol administration reduces the percentage of progression of stage 2 ROP to more-severe ROP from actual 36-37% [3] to zero, serial ophthalmologic evaluations are planned at different intervals according to the severity of ROP.

#### 2. Secondary endpoint

a. To evaluate the safety of propranolol treatment, cardiac and respiratory parameters (heart frequency, blood pressure, oxygen saturation, respiratory support) will be continuously monitored; blood samplings will be performed to check renal, liver and metabolic balance; urea plasma levels will be compared to evaluate the effect of propranolol on whole body protein metabolism

b. To evaluate if the number of laser treatments and vitrectomies is decreased in the newborns co-treated with propranolol.

c. To evaluate if propranolol improves the functional and structural outcome, visual function and ophthalmologic examination are planned at 40 weeks gestational age, 1, 4 1/2, 12, 18 and 24 months corrected age.

#### 3. Surrogate endpoint

Plasma concentration of proangiogenic markers (VEGF, the soluble forms of the tyrosine kinase receptors, sVEGFR-2 and sTie-2 and sE-selectin) will be measured in blood samples collected at randomization, before the beginning of treatment, and weekly for the first 3 weeks after randomization.

### n. Confidentiality

The participants' data collected during this trial will be kept confidential. Study staff will have access to the data as well as the participants' medical records as they pertain to this study. Published results will not contain any information that would identify individual participants.

## Discussion

The objective of this research is highly ambitious: to find a treatment simple, inexpensive, well tolerated and with few adverse effects, able to counteract one of the major complications of the prematurity. Our recent findings in the animal model indicate that the use of propranolol is promising and that this should be explored in human newborns. However, many uncertainties remain.

Firstly, it is unknown what dose of propranolol should be used in the newborns. We have demonstrated that, in OIR mice, proangiogenic factors were dose-dependently reduced by propranolol. We chose the dose of 2 mg/kg/day because is a low-dosage that usually does not produce adverse effects and because at this dosage propranolol is employed in the treatment of hemangiomas. However, it is likely that a higher dosage might be more effective or that a lower dosage might be safer. Secondly, it is currently unknown when propranolol should be started and how long it should be administered. Thirdly, a larger sample of patients could give more reliable answers about the effectiveness of this treatment.

In conclusion, this pilot study is the first research that explores the possible therapeutic role of beta_2 _blockers in ROP. Any favourable results of this research could open new perspectives and original scenarios about the treatment or the prevention of this and other proliferative retinopathies.

### Regulatory bodies

EUDRACT Number: 2004-002170-34

## List of abbreviations used

ROP: Retinopathy of prematurity; VEGF: Vascular Endothelial Growth Factor; IGF-1: Insulin-like Growth Factor-1; HIF: hypoxia-inducible factor; MAPK: mitogen-activated protein kinase; beta-AR: beta-adrenoreceptor; VEGFR-1: VEGF receptor 1; VEGFR-2: VEGF receptor 2; OIR: oxygen-induced retinopathy; DBS: dried blood spot.

## Competing interests

The authors declare that they have no competing interests.

## Authors' contributions

**LF **conceived the study, he is Chief Investigator, participated in the design and helped draft the manuscript. **GiC, PaF, GD and FM **participated in the design of the study and drafted the manuscript. **MD, VB, GlC, GA**, participated in the study design and are responsible for data collection. **SO, ALT, PiF, LP**, participated in the study design and are responsible for the ophthalmologic evaluations. **GLM **and **SM**, participated in the design of the study and are responsible for the determination of propranolol concentration on dried blood spots. **PB, CR, MDM**, performed the animal studies, participated in the design of the protocol and are responsible for the determination of plasma proangiogenic markers concentration in newborns **ARB, BI**, participated in the design of the study and reviewed the protocol **LR, GC, FT**, participated in the study design and are responsible for the visual function evaluations. **SF **participated in the study design and she is responsible for statistical analysis.

All authors contributed to the development of the protocol, and read and approved the final manuscript.

## Pre-publication history

The pre-publication history for this paper can be accessed here:

http://www.biomedcentral.com/1471-2431/10/83/prepub

## References

[B1] Gilbert C Retinopathy of prematurity: a global perspective of the epidemics, population of babies at risk and implications for control Early Hum Dev 2008 84 77 82 10.1016/j.earlhumdev.2007.11.009 18234457

[B2] Palmer EA Flynn JT Hardy RJ Phelps DL Phillips CL Schaffer DB Tung B Incidence and early course of retinopathy of prematurity. The Cryotherapy for Retinopathy of Prematurity Cooperative Group Ophthalmology 1991 98 1628 1640 1800923 10.1016/s0161-6420(91)32074-8

[B3] Good WV Hardy RJ Dobson V Palmer EA Phelps DL Quintos M Tung B Early Treatment for Retinopathy of Prematurity Cooperative Group The incidence and course of retinopathy of prematurity: findings from the early treatment for retinopathy of prematurity study Pediatrics 2005 116 15 23 10.1542/peds.2004-1413 15995025

[B4] Penn JS Madan A Caldwell RB Bartoli M Caldwell RW Hartnett ME Vascular endothelial growth factor in eye disease Prog Retin Eye Res 2008 27 331 371 10.1016/j.preteyeres.2008.05.001 18653375 PMC3682685

[B5] Madan A Penn JS Animal models of oxygen-induced retinopathy Front Biosci 2003 8 d1030 1043 10.2741/1056 12700061

[B6] West H Richardson WD Fruttiger M Stabilization of the retinal vascular network by reciprocal feedback between blood vessels and astrocytes Development 2005 132 1855 1862 10.1242/dev.01732 15790963

[B7] Smith LE Through the eyes of a child: understanding retinopathy through ROP the Friedenwald lecture Invest Ophthalmol Vis Sci 2008 49 5177 5182 10.1167/iovs.08-2584 18708611

[B8] Semenza GL Hydroxylation of HIF-1: oxygen sensing at the molecular level Physiology (Bethesda) 2004 19 176 182 15304631 10.1152/physiol.00001.2004

[B9] Pierce EA Foley ED Smith LE Regulation of vascular endothelial growth factor by oxygen in a model of retinopathy of prematurity Arch Ophthalmol 1996 114 1219 1228 8859081 10.1001/archopht.1996.01100140419009

[B10] Alon T Hemo I Itin A Pe'er J Stone J Keshet E Vascular endothelial growth factor acts as a survival factor for newly formed retinal vessels and has implications for retinopathy of prematurity Nat Med 1995 1 1024 1028 10.1038/nm1095-1024 7489357

[B11] Shweiki D Itin A Soffer D Keshet E Vascular endothelial growth factor induced by hypoxia may mediate hypoxia-initiated angiogenesis Nature 1992 359 843 845 10.1038/359843a0 1279431

[B12] Levy AP Levy NS Loscalzo J Calderone A Takahashi N Yeo KT Koren G Colucci WS Goldberg MA Regulation of vascular endothelial growth factor in cardiac myocytes Circ Res 1995 76 758 766 7728992 10.1161/01.res.76.5.758

[B13] Forsythe JA Jiang BH Iyer NV Agani F Leung SW Koos RD Semenza GL Activation of vascular endothelial growth factor gene transcription by hypoxia-inducible factor 1 Mol Cell Biol 1996 16 4604 4613 8756616 10.1128/mcb.16.9.4604 PMC231459

[B14] Ferrara N Davis-Smyth T The biology of vascular endothelial growth factor Endocr Rev 1997 18 4 25 10.1210/er.18.1.4 9034784

[B15] Pierce EA Avery RL Foley ED Aiello LP Smith LE Vascular endothelial growth factor/vascular permeability factor expression in a mouse model of retinal neovascularization Proc Natl Acad Sci USA 1995 92 905 909 10.1073/pnas.92.3.905 7846076 PMC42729

[B16] Jakeman LB Armanini M Phillips HS Ferrara N Developmental expression of binding sites and messenger ribonucleic acid for vascular endothelial growth factor suggests a role for this protein in vasculogenesis and angiogenesis Endocrinology 1993 133 848 859 10.1210/en.133.2.848 7688292

[B17] Robbins SG Rajaratnam VS Penn JS Evidence for upregulation and redistribution of vascular endothelial growth factor (VEGF) receptors flt-1 and flk-1 in the oxygen-injured rat retina Growth Factors 1998 16 1 9 10.3109/08977199809017487 9777366

[B18] Fujio Y Walsh K Akt mediates cytoprotection of endothelial cells by vascular endothelial growth factor in an anchorage-dependent manner J Biol Chem 1999 274 16349 16354 10.1074/jbc.274.23.16349 10347193 PMC3624707

[B19] Takahashi T Yamaguchi S Chida K Shibuya M A single autophosphorylation site on KDR/Flk-1 is essential for VEGF-A-dependent activation of PLC-gamma and DNA synthesis in vascular endothelial cells EMBO J 2001 20 2768 2778 10.1093/emboj/20.11.2768 11387210 PMC125481

[B20] Hellstrom A Perruzzi C Ju M Engstrom E Hard AL Liu JL Albertsson-Wikland K Carlsson B Niklasson A Sjodell L LeRoith D Senger DR Smith LE Low IGF-I suppresses VEGF-survival signaling in retinal endothelial cells: direct correlation with clinical retinopathy of prematurity Proc Natl Acad Sci USA 2001 98 5804 5808 10.1073/pnas.101113998 11331770 PMC33294

[B21] Hellström A Engström E Hård AL Albertsson-Wikland K Carlsson B Niklasson A Löfqvist C Svensson E Holm S Ewald U Holmström G Smith LE Postnatal serum insulin-like growth factor I deficiency is associated with retinopathy of prematurity and other complications of premature birth Pediatrics 2003 112 1016 1020 10.1542/peds.112.5.1016 14595040

[B22] Löfqvist C Andersson E Sigurdsson J Engström E Hård AL Niklasson A Smith LE Hellström A Longitudinal postnatal weight and insulin-like growth factor I measurements in the prediction of retinopathy of prematurity Arch Ophthalmol 2006 124 1711 1718 10.1001/archopht.124.12.1711 17159030

[B23] Smith LE Shen W Perruzzi C Soker S Kinose F Xu X Robinson G Driver S Bischoff J Zhang B Schaeffer JM Senger DR Regulation of vascular endothelial growth factor-dependent retinal neovascularization by insulin-like growth factor-1 receptor Nat Med 1999 5 1390 1395 10.1038/70963 10581081

[B24] Patz A Hoeck LE De La Cruz E Studies on the effect of high oxygen administration in retrolental fibroplasia. I. Nursery observations Am J Ophthalmol 1952 35 1248 1253 12976495 10.1016/0002-9394(52)91140-9

[B25] Kinsey VE Arnold HJ Kalina RE Stern L Stahlman M Odell G Driscoll JM Jr Elliott JH Payne J Patz A PaO2 levels and retrolental fibroplasia: a report of the cooperative study Pediatrics 1977 60 655 668 578921

[B26] Lucey JF Dangman B A reexamination of the role of oxygen in retrolental fibroplasia Pediatrics 1984 73 82 96 6419199

[B27] Bancalari E Flynn J Goldberg RN Bawol R Cassady J Schiffman J Feuer W Roberts J Gillings D Sim E Influence of transcutaneous oxygen monitoring on the incidence of retinopathy of prematurity Pediatrics 1987 79 663 669 3575019

[B28] STOP-ROP Multicenter Study Group Supplemental Therapeutic Oxygen for Prethreshold Retinopathy Of Prematurity (STOP-ROP), a randomized, controlled trial. I: primary outcomes Pediatrics 2000 105 295 310 10.1542/peds.105.2.295 10654946

[B29] Cryotherapy for Retinopathy of Prematurity Cooperative Group Contrast sensitivity at age 10 years in children who had threshold retinopathy of prematurity Arch Ophthalmol 2001 119 1129 1133 11483078 10.1001/archopht.119.8.1129

[B30] Dobson V Quinn GE Summers CG Hardy RJ Tung B Cryotherapy for Retinopathy of Prematurity Cooperative Group Visual acuity at 10 years in Cryotherapy for Retinopathy of Prematurity (CRYO-ROP) study eyes: effect of retinal residua of retinopathy of prematurity Arch Ophthalmol 2006 124 199 202 10.1001/archopht.124.2.199 16476889

[B31] Kivlin JD Biglan AW Gordon RA Dobson V Hardy RA Palmer EA Tung B Gilbert W Spencer R Cheng KP Buckley E Early retinal vessel development and iris vessel dilatation as factors in retinopathy of prematurity. Cryotherapy for Retinopathy of Prematurity (CRYO-ROP) Cooperative Group Arch Ophthalmol 1996 114 150 154 8573016 10.1001/archopht.1996.01100130144005

[B32] Early Treatment For Retinopathy Of Prematurity Cooperative Group Revised indications for the treatment of retinopathy of prematurity: results of the early treatment for retinopathy of prematurity randomized trial Arch Ophthalmol 2003 121 1684 1694 10.1001/archopht.121.12.1684 14662586

[B33] Good WV Early Treatment for Retinopathy of Prematurity Cooperative Group The Early Treatment for Retinopathy Of Prematurity Study: structural findings at age 2 years Br J Ophthalmol 2006 90 1378 1382 10.1136/bjo.2005.081166 16914473 PMC1857487

[B34] Early Treatment for Retinopathy of Prematurity Cooperative Group Good WV Hardy RJ Dobson V Palmer EA Phelps DL Tung B Redford M Final visual acuity results in the early treatment for retinopathy of prematurity study Arch Ophthalmol 2010 128 663 671 10.1001/archophthalmol.2010.72 20385926 PMC4162423

[B35] Ng EY Connolly BP McNamara JA Regillo CD Vander JF Tasman W A comparison of laser photocoagulation with cryotherapy for threshold retinopathy of prematurity at 10 years: part 1. Visual function and structural outcome Ophthalmology 2002 109 928 934 10.1016/S0161-6420(01)01017-X 11986099

[B36] Connolly BP Ng EY McNamara JA Regillo CD Vander JF Tasman W A comparison of laser photocoagulation with cryotherapy for threshold retinopathy of prematurity at 10 years: part 2. Refractive outcome Ophthalmology 2002 109 936 941 10.1016/S0161-6420(01)01015-6 11986101

[B37] Mintz-Hittner HA Kuffel RR Jr Intravitreal injection of bevacizumab (avastin) for treatment of stage 3 retinopathy of prematurity in zone I or posterior zone II Retina 2008 28 831 838 10.1097/IAE.0b013e318177f934 18536599

[B38] Quiroz-Mercado H Martinez-Castellanos MA Hernandez-Rojas ML Salazar-Teran N Chan RV Antiangiogenic therapy with intravitreal bevacizumab for retinopathy of prematurity Retina 2008 28 3 Suppl S19 25 10.1097/IAE.0b013e318159ec6b 18317339

[B39] Travassos A Teixeira S Ferreira P Regadas I Travassos AS Esperancinha FE Prieto I Pires G van Velze R Valido A Machado Mdo C Intravitreal bevacizumab in aggressive posterior retinopathy of prematurity Ophthalmic Surg Lasers Imaging 2007 38 233 237 17552391 10.3928/15428877-20070501-09

[B40] Robinson GS Ju M Shih SC Xu X McMahon G Caldwell RB Smith LE Nonvascular role for VEGF: VEGFR-1, 2 activity is critical for neural retinal development FASEB J 2001 15 1215 1217 11344092 10.1096/fj.00-0598fje

[B41] Léauté-Labrèze C Dumas de la Roque E Hubiche T Boralevi F Thambo JB Taïeb A Propranolol for severe hemangiomas of infancy N Engl J Med 2008 358 2649 2651 10.1056/NEJMc0708819 18550886

[B42] Sans V Dumas de la Roque E Berge J Grenier N Boralevi F Mazereeuw-Hautier J Lipsker D Dupuis E Ezzedine K Vergnes P Taïeb A Léauté-Labrèze C Propranolol for Severe Infantile Hemangiomas: Follow-Up Report Pediatrics 2009 in press 19706583 10.1542/peds.2008-3458

[B43] Lindgren I Altimiras J Chronic prenatal hypoxia sensitizes beta-adrenoceptors in the embryonic heart but causes postnatal desensitization Am J Physiol Regul Integr Comp Physiol 2009 297 R258 264 19458283 10.1152/ajpregu.00167.2009

[B44] Seya Y Fukuda T Isobe K Kawakami Y Takekoshi K Effect of norepinephrine on RhoA, MAP kinase, proliferation and VEGF expression in human umbilical vein endothelial cells Eur J Pharmacol 2006 553 54 60 10.1016/j.ejphar.2006.09.048 17070516

[B45] Fredriksson JM Lindquist JM Bronnikov GE Nedergaard J Norepinephrine induces vascular endothelial growth factor gene expression in brown adipocytes through a beta -adrenoreceptor/cAMP/protein kinase A pathway involving Src but independently of Erk1/2 J Biol Chem 2000 275 13802 13811 10.1074/jbc.275.18.13802 10788502

[B46] Guo K Ma Q Wang L Hu H Li J Zhang D Zhang M Norepinephrine-induced invasion by pancreatic cancer cells is inhibited by propranolol Oncol Rep 2009 22 825 830 19724861 10.3892/or_00000505

[B47] Yang EV Kim SJ Donovan EL Chen M Gross AC Webster Marketon JI Barsky SH Glaser R Norepinephrine upregulates VEGF, IL-8, and IL-6 expression in human melanoma tumor cell lines: implications for stress-related enhancement of tumor progression Brain Behav Immun 2009 23 267 275 10.1016/j.bbi.2008.10.005 18996182 PMC2652747

[B48] Yang EV Sood AK Chen M Li Y Eubank TD Marsh CB Jewell S Flavahan NA Morrison C Yeh PE Lemeshow S Glaser R Norepinephrine up-regulates the expression of vascular endothelial growth factor, matrix metalloproteinase (MMP)-2, and MMP-9 in nasopharyngeal carcinoma tumor cells Cancer Res 2006 66 10357 10364 10.1158/0008-5472.CAN-06-2496 17079456

[B49] Lutgendorf SK Cole S Costanzo E Bradley S Coffin J Jabbari S Rainwater K Ritchie JM Yang M Sood AK Stress-related mediators stimulate vascular endothelial growth factor secretion by two ovarian cancer cell lines Clin Cancer Res 2003 9 4514 4521 14555525

[B50] Sood AK Bhatty R Kamat AA Landen CN Han L Thaker PH Li Y Gershenson DM Lutgendorf S Cole SW Stress hormone-mediated invasion of ovarian cancer cells Clin Cancer Res 2006 12 369 375 10.1158/1078-0432.CCR-05-1698 16428474 PMC3141061

[B51] Thaker PH Han LY Kamat AA Arevalo JM Takahashi R Lu C Jennings NB Armaiz-Pena G Bankson JA Ravoori M Merritt WM Lin YG Mangala LS Kim TJ Coleman RL Landen CN Li Y Felix E Sanguino AM Newman RA Lloyd M Gershenson DM Kundra V Lopez-Berestein G Lutgendorf SK Cole SW Sood AK Chronic stress promotes tumor growth and angiogenesis in a mouse model of ovarian carcinoma Nat Med 2006 12 939 944 10.1038/nm1447 16862152

[B52] Drell TL 4th Joseph J Lang K Niggemann B Zaenker KS Entschladen F Effects of neurotransmitters on the chemokinesis and chemotaxis of MDA-MB-468 human breast carcinoma cells Breast Cancer Res Treat 2003 80 63 70 10.1023/A:1024491219366 12889599

[B53] Masur K Niggemann B Zanker KS Entschladen F Norepinephrine-induced migration of SW 480 colon carcinoma cells is inhibited by beta-blockers Cancer Res 2001 61 2866 2869 11306460

[B54] Palm D Lang K Niggemann B Drell TL Masur K Zaenker KS Entschladen F The norepinephrine-driven metastasis development of PC-3 human prostate cancer cells in BALB/c nude mice is inhibited by beta-blockers Int J Cancer 2006 118 2744 2749 10.1002/ijc.21723 16381019

[B55] Guimarães S Moura D Vascular adrenoceptors: an update Pharmacol Rev 2001 53 319 356 11356987

[B56] Iaccarino G Ciccarelli M Sorriento D Galasso G Campanile A Santulli G Cipolletta E Cerullo V Cimini V Altobelli GG Piscione F Priante O Pastore L Chiariello M Salvatore F Koch WJ Trimarco B Ischemic neoangiogenesis enhanced by beta2-adrenergic receptor overexpression: a novel role for the endothelial adrenergic system Circ Res 2005 97 1182 1189 10.1161/01.RES.0000191541.06788.bb 16239589

[B57] Praveen V Vidavalur R Rosenkrantz TS Hussain N Infantile hemangiomas and retinopathy of prematurity: possible association Pediatrics 2009 123 e484 489 10.1542/peds.2007-0803 19221153

[B58] Smith CP Sharma S Steinle JJ Age-related changes in sympathetic neurotransmission in rat retina and choroid Exp Eye Res 2007 84 75 81 10.1016/j.exer.2006.08.018 17074321

[B59] Steinle JJ Booz GW Meininger CJ Day JN Granger HJ Beta 3-adrenergic receptors regulate retinal endothelial cell migration and proliferation J Biol Chem 2003 278 20681 20686 10.1074/jbc.M300368200 12670949

[B60] Steinle JJ Cappocia FC Jr Jiang Y Beta-adrenergic receptor regulation of growth factor protein levels in human choroidal endothelial cells Growth Factors 2008 26 325 330 10.1080/08977190802442070 19021032

[B61] Walker RJ Steinle JJ Role of beta-adrenergic receptors in inflammatory marker expression in Müller cells Invest Ophthalmol Vis Sci 2007 48 5276 5281 10.1167/iovs.07-0129 17962483

[B62] Lashbrook BL Steinle JJ Beta-adrenergic receptor regulation of pigment epithelial-derived factor expression in rat retina Auton Neurosci 2005 121 33 39 10.1016/j.autneu.2005.05.006 15961351

[B63] Kubrusly RC Ventura AL de Melo Reis RA Serra GC Yamasaki EN Gardino PF de Mello MC de Mello FG Norepinephrine acts as D1-dopaminergic agonist in the embryonic avian retina: late expression of beta1-adrenergic receptor shifts norepinephrine specificity in the adult tissue Neurochem Int 2007 50 211 218 10.1016/j.neuint.2006.08.004 17014930

[B64] Smith LE Wesolowski E McLellan A Kostyk SK D'Amato R Sullivan R D'Amore PA Oxygen-induced retinopathy in the mouse Invest Ophthalmol Vis Sci 1994 35 101 111 7507904

[B65] Chen J Smith LE Retinopathy of prematurity Angiogenesis 2007 10 133 140 10.1007/s10456-007-9066-0 17332988

[B66] Ristori C Filippi L Dal Monte M Martini D Cammalleri M Fortunato P la Marca G Fiorini P Bagnoli P Role of the adrenergic system in a mouse model of oxygen-induced retinopathy: antiangiogenic effects of beta-adrenoreceptor blockade Invest Ophthalmol Vis Sci in press 10.1167/iovs.10-5536 20739470

[B67] Frishman WH Beta-adrenergic receptor blockers. Adverse effects and drug interactions Hypertension 1988 11 II21 29 2895072 10.1161/01.hyp.11.3_pt_2.ii21

[B68] Gardner LI Is propranolol alone really beneficial in neonatal thyrotoxicosis? Bradycardia and hypoglycemia evoke the doctrine of primum non nocere Am J Dis Child 1980 134 819 820 6998280 10.1001/archpedi.1980.02130210003001

[B69] Newman TJ Virnig NL Athinarayanan PR Complications of propranolol use in neonatal thyrotoxicosis Am J Dis Child 1980 134 707 708 7395835 10.1001/archpedi.1980.02130190073021

[B70] Ricci D Cesarini L Groppo M De Carli A Gallini F Serrao F Fumagalli M Cowan F Ramenghi LA Anker S Mercuri E Mosca F Early assessment of visual function in full term newborns Early Hum Dev 2008 84 107 113 10.1016/j.earlhumdev.2007.03.010 17513071

[B71] Ricci D Cesarini L Gallini F Serrao F Leone D Baranello G Cota F Pane M Brogna C De Rose P Vasco G Alfieri P Staccioli S Romeo DM Tinelli F Molle F Lepore D Baldascino A Ramenghi LA Torrioli MG Romagnoli C Cowan F Atkinson J Cioni G Mercuri E Cortical visual function in preterm infants in the first year J Pediatr 2010 156 550 555 10.1016/j.jpeds.2009.10.042 20056237

[B72] la Marca G Malvagia S Filippi L Luceri F Moneti G Guerrini R A new rapid micromethod for the assay of phenobarbital from dried blood spots by LC-tandem mass spectrometry Epilepsia 2009 50 2658 2662 10.1111/j.1528-1167.2009.02204.x 19682026

[B73] la Marca G Malvagia S Filippi L Fiorini P Innocenti M Luceri F Pieraccini G Moneti G Francese S Dani FR Guerrini R Rapid assay of topiramate in dried blood spots by a new liquid chromatography-tandem mass spectrometric method J Pharm Biomed Anal 2008 48 1392 1396 10.1016/j.jpba.2008.09.025 18980824

[B74] Pieh C Krüger M Lagrèze WA Gimpel C Buschbeck C Zirrgiebel U Agostini HT Plasma sE-selectin in premature infants: a possible surrogate marker of retinopathy of prematurity Invest Ophthalmol Vis Sci 2010 51 3709 3713 10.1167/iovs.09-4723 20181841

[B75] Pieh C Agostini H Buschbeck C Krüger M Schulte-Mönting J Zirrgiebel U Drevs J Lagrèze WA VEGF-A, VEGFR-1, VEGFR-2 and Tie2 levels in plasma of premature infants: relationship to retinopathy of prematurity Br J Ophthalmol 2008 92 689 693 10.1136/bjo.2007.128371 18408080

[B76] Glantz SA Primer of Biostatistics 2005 6 New York: McGraw-Hill

[B77] Koehne P Hochhaus F Felderhoff-Mueser U Ring-Mrozik E Obladen M Buhrer C Vascular endothelial growth factor and erythropoietin concentrations in cerebrospinal fluid of children with hydrocephalus Childs Nerv Syst 2002 18 137 141 10.1007/s00381-002-0567-2 11981620

[B78] Heep A Stoffel-Wagner B Bartmann P Benseler S Schaller C Groneck P Obladen M Felderhoff-Mueser U Vascular endothelial growth factor and transforming growth factor-beta1 are highly expressed in the cerebrospinal fluid of premature infants with posthemorrhagic hydrocephalus Pediatr Res 2004 56 768 776 10.1203/01.PDR.0000141524.32142.53 15319463

